# Assessment of right ventricular systolic function using speckle tracking strain imaging in patients with severe tricuspid regurgitation: a validation study with cardiac magnetic resonance

**DOI:** 10.1186/s44348-024-00015-4

**Published:** 2024-08-07

**Authors:** Inki Moon, Soongu Kwak, MinKwan Kim, Seung-Pyo Lee, Hyung-Kwan Kim, Yong-Jin Kim, Jun-Bean Park

**Affiliations:** 1 Department of Internal Medicine, Soonchunhyang University Bucheon Hospital, Soonchunhyang University College of Medicine, Bucheon, Republic of Korea; 2 Division of Cardiology, Department of Internal Medicine, Seoul National University Hospital, Seoul, Republic of Korea; 3 Department of Internal Medicine, Seoul National University College of Medicine, Seoul, Republic of Korea; 4 Department of Internal Medicine, Yongin Severance Hospital, Yonsei University College of Medicine, Yongin, Republic of Korea

**Keywords:** Echocardiography, Right ventricular dysfunction, Tricuspid valve insufficiency, Global longitudinal strain

## Abstract

**Background:**

Right ventricular (RV) systolic dysfunction is an established prognostic factor in patients with severe tricuspid regurgitation (TR). However, accurate assessment of RV systolic function using conventional echocardiography remains challenging. We investigated the accuracy of strain measurement using speckle tracking echocardiography (STE) for evaluating RV systolic function in patients with severe TR.

**Methods:**

We included consecutive patients with severe TR who underwent echocardiography and cardiac magnetic resonance imaging (CMR) within 30 days between 2011 and 2023. Two-dimensional STE was used to measure RV free wall longitudinal strain (RVFWLS) and global longitudinal strain (RVGLS). These values were compared with the RV ejection fraction (RVEF) from CMR. RV systolic dysfunction was defined as a CMR-derived RVEF < 35%.

**Results:**

A total of 87 patients with severe TR were identified during the study period. Among echocardiographic RV strain measurements, RVFWLS was the best correlate of CMR-derived RVEF (*r* = –0.37, *P* < 0.001), followed by RVGLS (*r* = –0.27, *P* = 0.012). Receiver operating characteristic (ROC) curve analysis revealed that RVFWLS provided better discrimination of RV systolic dysfunction, yielding an area under the ROC curve (AUC) of 0.770 (95% confidence interval [CI], 0.696–0.800) than RV fractional area change (AUC, 0.615; 95% CI, 0.500–0.859).

**Conclusions:**

In patients with severe TR, STE-derived RVFWLS showed the best correlation with RVEF on CMR and displayed superior discrimination of RV systolic dysfunction compared with the RV fractional area change. This study suggests the potential usefulness of STE in assessing RV systolic function in this population.

**Supplementary Information:**

The online version contains supplementary material available at 10.1186/s44348-024-00015-4.

## Background

Severe tricuspid regurgitation (TR) has been repeatedly implicated as an independent risk factor for adverse cardiovascular outcomes [1, 2]. Furthermore, as the association between TR and advancing age has been widely recognized, the importance of detection and management of TR bas become increasingly clear with the aging population [3]. The presence of severe TR leads to right ventricular (RV) volume overload, which results in progressive RV enlargement and further worsening of TR [1, 2]. In the course of this vicious cycle, the main hemodynamic consequence is RV systolic dysfunction, which is an independent predictor of adverse clinical outcomes in patients with severe TR, even following successful TR surgery [4]. A thorough echocardiographic follow-up of RV systolic function is thus strongly recommended in patients with severe TR [5]. However, the RV has a more complex geometry compared to the left ventricle (LV), which poses challenges for accurate and reproducible echocardiographic measures of RV volume and function. Moreover, patients with severe TR have a dilated RV, hampering visualization of the entire RV cavity. In this regard, cardiac magnetic resonance imaging (CMR) currently represents the gold standard for RV volumetric assessment [4–6]. However, the use of CMR is limited in daily practice because of its high cost and time-consuming procedures; therefore, advanced echocardiographic techniques such as strain may be useful alternative imaging tools.

LV strain measured using two-dimensional (2D) speckle tracking echocardiography (STE) is an accepted parameter that reflects LV myocardial function and has transitioned from research to clinical use. Although the role of RV strain assessment is relatively not well established, several studies have demonstrated its usefulness in assessing RV systolic function in various pathologies, such as pulmonary hypertension [7] and ischemic heart disease [8]. However, conventional echocardiography-based RV parameters in patients with severe TR have presented moderate performance in the detection of RV systolic dysfunction [9, 10], possibly owing to their susceptibility to loading conditions and reliance on geometric assumptions of complex RV morphology. Speckle tracking–derived RV strain has been proven to be less affected by loading conditions and avoid geometrical assumptions by directly measuring myocardial deformation, which is advantageous for overcoming the limitations of conventional RV parameters. Therefore, in the present study, we aimed to investigate the accuracy of RV strain in assessing RV systolic function in comparison with CMR as the reference standard in patients with severe TR.

## Methods

### Ethics statement

The study protocol conformed to the ethical guidelines of the Declaration of Helsinki and was approved by the Institutional Review Board of Seoul National University Hospital (No. 1009–014-331). Informed consent was waived due to the retrospective nature of the study.

### Study population and characteristics

The cohort for this retrospective study included isolated patients with severe TR who underwent both echocardiography and CMR within 30 days and were recruited from a single tertiary hospital between August 2011 and March 2023. All patients met the following echocardiographic criteria for severe TR: (1) TR jet area > 30% of the right atrial area; (2) inadequate coaptation of the tricuspid leaflets; and (3) systolic flow reversal in the hepatic veins [4, 5, 8]. We excluded patients with severe TR caused by left-sided heart disease and pulmonary hypertension. We also excluded patients with moderate or severe left-sided valve disease to include only those with severe TR as a single significant lesion. Additionally, patients were excluded if image quality was insufficient for the measurement of RV strain. Ultimately, 87 patients with isolated severe TR were included in the analysis. We collected clinical data, including New York Heart Association (NYHA) Functional Classification, at the time of echocardiography. Baseline blood test results were obtained, and the glomerular filtration rate was estimated using the Chronic Kidney Disease Epidemiology Collaboration (CKD-EPI) equation.

### Echocardiographic measurements

Comprehensive echocardiographic studies were performed in all patients by experienced clinical sonographers using commercially available instruments (General Electric Healthcare, Philips, and Siemens). The 2D, M-mode, and Doppler measurements were obtained using standard techniques and procedures according to the guidelines from the American Society of Echocardiography [11]. RV end-diastolic and end-systolic areas were obtained from the RV-focused apical four chamber view, and the RV fractional area change (FAC) was calculated as “[(RV end-diastolic area − RV end-systolic area) / RV end-diastolic area].” The pulmonary artery systolic pressure was estimated from the peak TR velocity. All images used to analyze the RV peak systolic longitudinal strain were recorded at a minimum of 50 fps to ensure reliable analysis. To measure RV strain, vendor-independent 2D speckle tracking software (Image-Arena, TomTec Imaging Systems GmbH) was used by an experienced sonographer who was blinded to the clinical information of the patients. In brief, The RV end-diastolic endocardial border was manually traced along the RV septal and RV free wall from an RV-focused apical four chamber view (Supplementary Fig. 1, Additional File 1). The software automatically tracked speckles along the RV endocardial border and myocardium through the cardiac cycle [12]. We used RV free wall longitudinal strain (RVFWLS) as the primary method for measuring RV strain, consistent with the current guidelines. RVFWLS was calculated as the average of the three RV lateral segments (basal, mid, and apical), excluding the three septal segments, whereas the RV global longitudinal strain (RVGLS) was calculated as the average of all six segments.

### Cardiac magnetic resonance imaging

Standard CMR was performed using a 1.5-T system (Sonata Magnetom, Siemens) equipped with a cardiac phased-array receiver coil. The same imaging unit was used for all patients throughout the study period. Steady-state free-precession cine images were obtained with a firm breath-hold to visualize ventricular motion. We acquired entire short-axis images at a 6-mm interval with a 4-mm intersection gap from the valve plane to the apex, thereby including the whole ventricular volume. These images were used to perform volumetric analysis as described previously [4]. Both ventricular end-diastolic and end-systolic volumes, stroke volumes, cardiac output, and ejection fractions (EFs) were measured using dedicated software (QMASS MR ver. 6.2.1, Medis). We defined RV systolic dysfunction as an RVEF of < 35%, according to previous publications [13–15]. Ventricular volumes and cardiac output were normalized to the body surface area. To quantify the net pulmonary blood volume ejected by the RV, velocity-encoded cine CMR with retrospective electrocardiographic gating and free breathing was performed in a plane perpendicular to the left and right pulmonary arteries [4, 16]. Specialized software (Argus, Siemens) was used to analyze the flow profiles of the velocity-encoded cine CMR images. The contours of each pulmonary artery were automatically delineated on the magnitude and velocity map images of all reconstructed phases, with manual correction when required. All CMR images were analyzed by expert radiologists who were blinded to the strain results.

### Statistical analysis

The continuous variables are presented as either mean ± standard deviation or median (interquartile range), and the categorical variables are presented as percentages. The Student t-test was used to compare normally distributed continuous variables, whereas the Wilcoxon rank sum test was used to compare non-normally distributed continuous variables. The chi-square test or Fisher exact test was used to compare categorical variables between the two groups. Statistical significance was set at a two-sided *P*-value < 0.05. The correlation between RV strain values obtained by echocardiography and CMR parameters was assessed using Spearman rank correlation coefficient (ρ). Receiver operating characteristic (ROC) curve analysis was performed, and areas under the curve (AUCs) were calculated to evaluate the discriminative ability of RV strain and RVFAC for RV systolic dysfunction, which was defined as RVEF < 35% on CMR. The DeLong test was used to compare the two ROC curves. All statistical analyses were performed using R ver. 3.4.3 (R Foundation for Statistical Computing).

## Results

The baseline clinical characteristics of 87 patients with isolated severe TR are presented in Table 1. The mean age of the cohort was 62.8 ± 11.3 years, with the majority being women (65 of 87 patients, 74.7%) and having atrial fibrillation (74 of 87 patients, 85.1%). More than half of the patients received digoxin and diuretics. No significant abnormalities in the laboratory results were observed.

**Table 1:** Baseline characteristics of the study population

Characteristic	Value (*n* = 87)
Age (yr)	62.8 ± 11.3
Female sex	65 (74.7)
Body surface area (m^2^)	1.57 ± 0.18
Systolic blood pressure (mmHg)	119.5 ± 16.5
Diastolic blood pressure (mmHg)	68.3 ± 10.9
Atrial fibrillation	74 (85.1)
Medication	
β-blockers	25 (28.7)
RAS blockade	23 (26.4)
Digoxin	35 (40.2)
Loop diuretics	59 (67.8)
Spironolactone	53 (60.9)
Thiazide	20 (23.0)
Laboratory test	
Hemoglobin (g/dL)	12.3 ± 2.2
Platelet count (× 10^3^/mL)	145.3 ± 60.0
Total protein (g/dL)	7.2 ± 0.8
Albumin (g/dL)	4.1 ± 0.5
Total cholesterol (mg/dL)	148.7 ± 38.2
Blood urea nitrogen (mg/dL)	23.0 ± 12.5
Creatinine (mg/dL)	0.9 (0.8–1.2)
Glomerular filtration rate (mL/min/1.73 m^2^)	74.0 (51.0–84.5)

Values are presented as mean ± standard deviations, number (%), or median (interquartile range)

*RAS* Renin-angiotensin system

Table 2 outlines the echocardiographic and CMR measurements of the patients with isolated severe TR. Among the echocardiographic parameters measured, the mean LVEF was 57.5% ± 6.2% and the mean estimated pulmonary artery systolic pressure was 39.2 ± 10.6 mmHg. The mean values of tricuspid annular diameter, indexed RV end-diastolic and end-systolic areas, and RVFAC were 46.8 ± 11.5 mm, 20.7 ± 5.1 cm^2^/m^2^, 10.2 ± 4.0 cm^2^/m^2^, and 44.6% ± 9.3%, respectively. With regard to RV strain analysis, the mean values of RVFWLS and RVGLS were –29.4% ± 6.0% and –25.4 ± 5.4%, respectively. Of the CMR parameters assessed, the mean LVEF was 56.5% ± 10.5%, which was similar to that measured by echocardiography. The mean values of RV end-diastolic volume (RVEDV), RV end-systolic volume (RVESV), and indexed RVEDV and RVESV were 280.5 ± 108.2 mL, 150.1 ± 69.1 mL, 179.7 ± 67.8 mL/m^2^, and 96.3 ± 44.5 mL/m^2^, respectively. The mean RVEF estimated by CMR was 47.5% ± 9.3%, and eight patients had with RV systolic dysfunction (RVEF < 35%).

**Table 2:** Echocardiographic and CMR measurements of the study population

Variable	Total (*n* = 87)
Echocardiographic measurement	
Indexed RV end-diastolic area (cm^2^/m^2^)	20.7 ± 5.1
Indexed RV end-systolic area (cm^2^/m^2^)	10.2 ± 4.0
RV fractional area change (%)	44.6 ± 9.3
RV free wall longitudinal strain (%)	–29.4 ± 6.0
RV global longitudinal strain (%)	–25.4 ± 5.4
Tricuspid annulus diameter (mm)	46.8 ± 11.5
LV end-diastolic diameter (mm)	46.2 ± 6.1
LV end-systolic diameter (mm)	29.8 ± 4.4
LV ejection fraction (%)	57.5 ± 6.2
Pulmonary artery systolic pressure (mmHg)	39.2 ± 10.6
CMR measurement	
RV end-diastolic volume (mL)	280.5 ± 108.2
RV end-systolic volume (mL)	150.1 ± 69.1
Indexed RV end-diastolic volume (mL/m^2^)	179.7 ± 67.8
Indexed RV end-systolic volume (mL/m^2^)	96.3 ± 44.5
RV ejection fraction (%)	47.5 ± 9.3
RV stroke volume (mL)	130.4 ± 50.4
RV cardiac output (L/min)	8.9 ± 3.1
RV dysfunction	8 (9.2)
LV end-diastolic volume (mL)	132.5 ± 44.6
LV end-systolic volume (mL)	59.2 ± 25.9
LV ejection fraction (%)	56.5 ± 10.5
LV stroke volume (mL)	75.1 ± 25.0
LV cardiac output (L/min)	5.2 ± 1.8

Values are presented as mean ± standard deviation or number (%)

*CMR* Cardiac magnetic resonance imaging, *RV* Right ventricular, *LV* Left ventricular

**Fig. 1:**
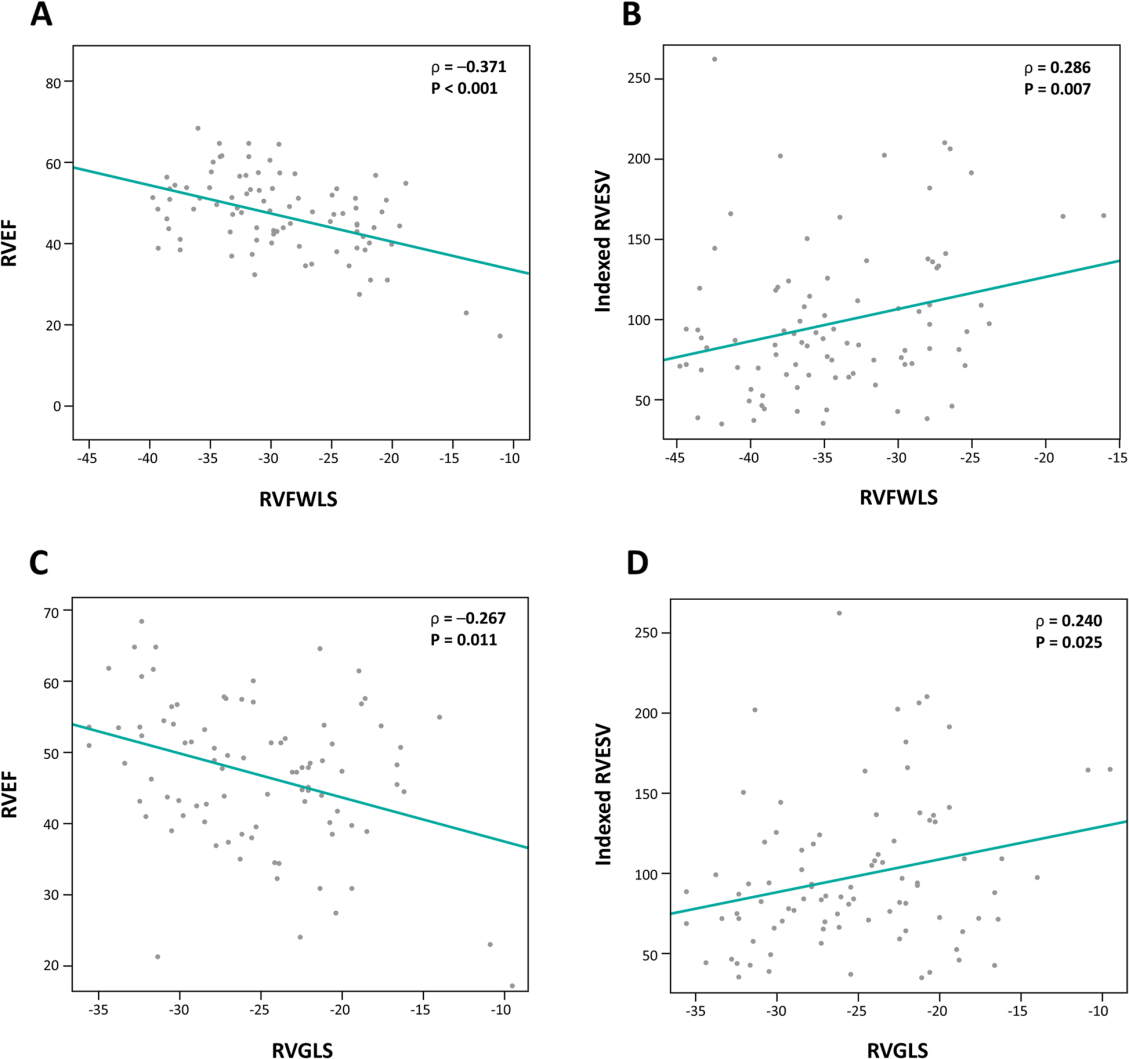
Spearman correlation analysis of speckle tracking echocardiography derived right ventricular (RV) strain and cardiac magnetic resonance imaging (CMR)-derived RV parameters. The RV ejection fraction (RVEF) measured using CMR was related to (**A**) RV free wall longitudinal strain (RVFWLS) and (**C**) RV wall global longitudinal strain (RVGLS), as measured using two-dimensional speckle tracking echocardiography. The indexed RV end-systolic volume (RVESV) is also related to (**B**) RVFWLS and (**D**) RVGLS

Figure 1 illustrates the correlation between the RV strain parameters measured by STE and the RV parameters measured by CMR. There was a significant correlation between RVFWLS and RVEF (*ρ* = –0.371, *P* < 0.001) (Fig. 1A), between RVFWLS and indexed RVESV (*ρ* = 0.286, *P* = 0.007) (Fig. 1B), between RVGLS and RVEF (*ρ* = –0.267, *P* = 0.012) (Fig. 1C), and between RVGLS and indexed RVESV (*ρ* = 0.240, *P* = 0.025) (Fig. 1D). The results of the correlation analysis between other echocardiographic and CMR measures of RV size and function are summarized in Supplementary Table 1 (Additional File 1). In comparison with RVFWLS, RVFAC measured by conventional echocardiography was less strongly correlated with RVEF (*ρ* = 0.282, *P* = 0.008) and indexed RVESV (*ρ* = –0.200, *P* = 0.063) by CMR. However, indexed RV end-systolic area (RVESA) by conventional echocardiography showed a good correlation with RVEF (*ρ* = –0.399, *P* < 0.001) and indexed RVESV (*ρ* = –0.730, *P* < 0.001).

**Fig. 2:**
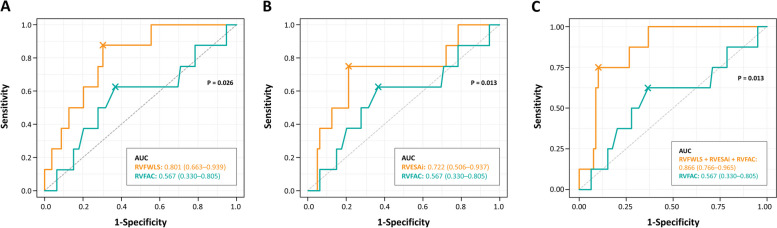
Receiver operating characteristic curve analysis of right ventricular free wall longitudinal strain (RVFWLS), indexed RV end-systolic area (RVESAi), and RV fractional area change (RVFAC) for RV systolic dysfunction. AUC, area under the receiver operating characteristic curve

ROC analysis showed that RVFWLS provided moderately good discrimination of RV systolic dysfunction (RVEF < 35%), yielding an AUC of 0.801 (95% confidence interval [CI], 0.663–0.939). The AUC of RVGLS was 0.742 (95% CI, 0.615–0.869) and the AUC of indexed RVESA was 0.722 (95% CI, 0.506–0.937), which was smaller but not significantly different from RVFWLS. When comparing the AUCs for RVFWLS and RVFAC, RVFWLS had significantly better discrimination of RV systolic dysfunction than RVFAC (AUC, 0.801 vs. 0.567; *P* = 0.026) (Fig. 2A). Indexed RVESA also showed better discriminatory performance in detecting RV systolic dysfunction than did RVFAC (AUC, 0.722 vs. 0.567; *P* = 0.013) (Fig. 2B). The AUC of the model created by combining RVFWLS, indexed RVESA, and RVFAC was significantly larger than that of RVFAC alone (AUC, 0.866 vs. 0.567; *P* = 0.002) (Fig. 2C).

## Discussion

The main findings of our study were as follows: (1) RVFWLS on echocardiography showed a significant correlation with RVEF on CMR in patients with severe isolated TR; (2) RVFWLS provided better discrimination of RV systolic dysfunction determined by CMR than RVFAC, a traditional echocardiographic parameter of RV systolic function; and (3) the combined assessment of RVFWLS, indexed RVESA, and RVEF improved the discriminatory value of RV systolic dysfunction over RVEF alone. The assessment of ventricular systolic function is a crucial step in determining the need for corrective surgery in asymptomatic patients with severe valvular heart disease [5]. Accurate assessment of RV systolic function is also important in patients with severe TR, particularly considering that the symptoms associated with this condition, such as weakness and fatigue, can be ambiguous. However, conventional echocardiography has been limited in assessing RV systolic function in these patients [1, 9, 17]. Although CMR can overcome this limitation, its technical complexity and high cost hamper its widespread clinical use in patients with severe TR, who require periodic monitoring of RV parameters owing to the progressive nature of RV systolic dysfunction. Therefore, our results suggest that RV strain indices have promising potential for the accurate and practical assessment of RV systolic function in patients with severe TR.

Conventional echocardiography provides valuable data for treatment decision-making, including determining candidacy for surgical or interventional procedures, in patients with mitral regurgitation (MR). Specifically, in asymptomatic patients with severe MR, surgery is recommended if LVEF ≤ 60% and/or LV end-systolic diameter ≥ 40 mm. A similar approach may be appropriate in patients with severe TR, which has a hemodynamic basis similar to MR. However, unlike the utility of LVEF assessment or monitoring in patients with MR, the utility of 2D echocardiographic indices for RV systolic function is less clear in patients with TR. Tricuspid annular plane systolic excursion (TAPSE) has been widely used to estimate RV systolic function based on anatomical observations that show the predominant longitudinal orientation of RV muscle fibers. Although TAPSE has proven to be a significant prognostic factor in patients with pulmonary hypertension [18] and heart failure [19], it has not demonstrated the same predictive value in patients with severe TR [9, 10, 17]. Furthermore, TAPSE in the setting of severe TR showed no significant correlation with RVEF on CMR [20], and its reproducibility is limited due to Doppler angle dependency, which is exaggerated by the enlarged RV in patients with severe TR. Another conventional echocardiographic index, RVFAC, has only fair reproducibility. In the process of tracing the RV area that is required to calculate RVFAC, it is important to accurately include the trabeculae in the RV cavity, which results in increased measurement time and reduced reproducibility [21]. More importantly, although RVFAC has an advantage in assessing global systolic function of the RV compared with TAPSE, optimal visualization of the entire RV is a prerequisite for its measurement [21]. As patients with severe TR frequently have markedly enlarged RV, this prerequisite makes it practically difficult to reliably measure RVFAC in a significant number of patients. RVFAC could not be measured in 14 patients (15.2%) in our study. Regarding the prognostic role of RVFAC in severe TR, a previous study suggested incremental risk stratification with early postoperative RVFAC in patients undergoing corrective TR surgery [22]. However, preoperative RVFAC did not improve risk prediction in this patient population [4], substantially limiting its clinical usefulness in determining the optimal surgical timing. These drawbacks of traditional 2D echocardiography warrant consideration of alternative modalities for assessing RV systolic function in these patients [23, 24]. Because of its superior accuracy and reproducibility, CMR is recommended as the gold standard for evaluating RV volumes and function in patients with severe TR, when available [5]. However, the disadvantages of CMR are its limited availability, high cost, and the high level of scanning expertise required for image acquisition. Another important limitation of CMR is that this imaging modality cannot be used in patients with intracardiac devices due to safety concerns and in patients with dyspnea due to the need for breath-holding during acquisition for up to 5 to 8 s [25], both of which are commonly encountered situations in the assessment of severe TR.

RV strain analysis has several advantages that make it the preferred imaging modality for assessing RV systolic function in clinical practice. It has been reported that speckle tracking–derived RV strain is relatively angle-independent compared to other echocardiographic parameters reflecting RV longitudinal systolic function, including TAPSE and tricuspid lateral annular systolic velocity [21]. In this regard, it is not surprising that there have been numerous studies investigating the correlation between RV strain measured using STE and RVEF measured by CMR. However, there was a paucity of evidence assessing this correlation in patients with severe TR. The present study showed a modest correlation between the values of STE-derived RV strain and CMR-derived RVEF. Furthermore, we explored the discriminating ability of RVFWLS for RV systolic dysfunction as indicated by RVEF on CMR. On the other hand, there have been conflicting results regarding which of RVFWLS and RVGLS is a better indicator of RV systolic function [26–28]. However, several studies showed that RVFWLS was better correlated than RVGLS with RVEF on CMR [12]. Furthermore, in patients with severe TR, there are several studies suggesting that RVFWLS has superior prognostic predictive ability compared to RVGLS, which are in line with our results [27, 29]. Both 3D echocardiography and CMR have advantages in terms of prognostic capability, however they can be challenging to be integrated in clinical routine, due to the relatively long time required to acquire imaging data and high costs. Unlike these imaging modalities, RV strain is a parameter that can be readily measured during daily echocardiographic examinations. Therefore, incorporating RV strain analysis could enhance the applicability of serial 2D echocardiography in managing patients with severe TR. Additionally, we found an improvement in the discrimination of RV systolic dysfunction by combining the RV strain, indexed RVESA, and RVFAC. This finding suggests that in patients with severe TR, even if the RVFAC is normal, reduced RV strain and/or increased RV size may indicate the possibility of RV systolic dysfunction. Taken together, RV strain may be a useful echocardiographic marker for monitoring RV systolic function and a potential gatekeeper for CMR as a confirmatory test for diagnosing RV systolic dysfunction in patients with severe TR. The use of RV strain as part of a monitoring strategy of TR patients with TR could enable the early detection of RV systolic dysfunction for optimal timing of corrective surgery.

This study had several limitations. First, this was a retrospective, single-center study conducted on a relatively small cohort of patients with severe TR. The limited sample size was attributed to the low incidence of severe TR and the need to select patients who underwent echocardiography and CMR at appropriate intervals. Furthermore, a highly imbalanced dataset with few cases of RV dysfunction might lead to the overestimation of the performance of ROC curve analysis. However, our study is the first to explore the role of RV strain derived from STE in the assessment of RV systolic function compared to CMR in this population. Further studies with larger cohorts are required to confirm these findings. Second, the issue of reproducibility arises when values measured by different imaging modalities are compared. However, our previous studies showed that assessments of RV strain using echocardiography and RVEF using CMR had excellent intraobserver and interobserver reliability [4, 29]. Notably, in our previous study where 86% of the patients had atrial fibrillation, the intraobserver and interobserver reproducibility was high in the measurement of RVFWLS (intraobserver reproducibility intraclass correlation coefficient [ICC], 0.98 [95% CI, 0.96–0.99]; interobserver reproducibility ICC, 0.88 [95% CI, 0.78–0.93]) [29]. When we analyzed 28 randomly selected patients from the present study, intraobserver and interobserver reproducibility for RVFWLS was similar with previous result (intraobserver reproducibility ICC, 0.93 [95% CI, 0.84–0.95]; interobserver reproducibility ICC, 0.85 [95% CI, 0.67–0.93]). Third, data on TAPSE and peak systolic tricuspid annular velocity were not available in this study; thus, we could not assess the correlation between these echocardiographic parameters and RVEF using CMR and their discriminative ability for RV systolic dysfunction, defined as CMR-derived RVEF < 35%. However, it has been reported that, in patients with severe TR, both TAPSE and peak systolic tricuspid annular velocity have no significant correlation to RVEF on CMR [20]. Finally, it is important to consider potential intervendor differences in RV strain measurements. We used a vendor-independent program to mitigate this concern.

## Conclusions

Speckle tracking–derived RV strain showed a significant correlation with RVEF measured using CMR and provided significant discrimination for RV dysfunction in patients with severe TR. Therefore, this strain analysis may assist in monitoring RV systolic function and assessing the need for further investigations such as CMR during the follow-up of these patients.

### Supplementary Information

Additional file 1. Supplementary Fig. 1 and Supplementary Table 1.

## Data Availability

The datasets used and/or analyzed during the current study are available from the corresponding author on reasonable request.

## Abbreviations

D: Dimensional
AUC: Area under the curve
CKD-EPI: Chronic Kidney Disease Epidemiology Collaboration
CI: Confidence interval
CMR: Cardiac magnetic resonance imaging
EF: Ejection fraction
FAC: Fractional area change
ICC: Intraclass correlation coefficient
LV: Left ventricle
LVEF: Left ventricular ejection fraction
MR: Mitral regurgitation
NYHA: New York Heart Association
ROC: Receiver operating characteristic
RV: Right ventricular
RVEDV: Right ventricular end-diastolic volume
RVEF: Right ventricular ejection fraction
RVESA: Right ventricular end-systolic area
RVESV: Right ventricular end-systolic volume
RVFAC: Right ventricular fractional area change
RVFWLS: Right ventricular free wall longitudinal strain
RVGLS: Right ventricular global longitudinal strain
STE: Speckle tracking echocardiography
TAPSE: Tricuspid annular plane systolic excursion
TR: Tricuspid regurgitation
