# Supplementary material for: Assessment of right ventricular systolic function using speckle tracking strain imaging in patients with severe tricuspid regurgitation: a validation study with cardiac magnetic resonance
Source: J Cardiovasc Imaging. 2024 Aug 7;32:22. doi: 10.1186/s44348-024-00015-4 (PMC11304584; doi:10.1186/s44348-024-00015-4)
Supplement: Supplementary file 1 — Additional file 1. Supplementary Fig. 1 and Supplementary Table 1. [file 44348_2024_15_MOESM1_ESM.docx]

**Supplementary Table 1. Correlation analysis between echocardiographic- and CMR-derived measurements**

| **Parameter** |  | |
| --- | --- | --- |
|  | **ρ** | ***P*-value** |
| **Indexed RVEDV by CMR** |  |  |
| Indexed RV end-diastolic area, cm^2^/m^2^ | 0.672 | <0.001 |
| Indexed RV end-systolic area, cm^2^/m^2^ | 0.704 | <0.001 |
| RV fractional area change, % | -0.130 | 0.23 |
| RV free wall longitudinal strain | 0.179 | 0.098 |
| RV global longitudinal strain | 0.184 | 0.087 |
| **Indexed RVESV by CMR** |  |  |
| Indexed RV end-diastolic area, cm^2^/m^2^ | 0.605 | <0.001 |
| Indexed RV end-systolic area, cm^2^/m^2^ | 0.730 | <0.001 |
| RV fractional area change, % | -0.200 | 0.063 |
| RV free wall longitudinal strain | 0.286 | 0.007 |
| RV global longitudinal strain | 0.240 | 0.025 |
| **RVEF by CMR** |  |  |
| Indexed RV end-diastolic area, cm^2^/m^2^ | -0.018 | 0.904 |
| Indexed RV end-systolic area, cm^2^/m^2^ | -0.399 | <0.001 |
| RV fractional area change, % | 0.282 | 0.008 |
| RV free wall longitudinal strain | -0.371 | <0.001 |
| RV global longitudinal strain | -0.267 | 0.012 |

ρ indicates Spearman ρ.

CMR, cardiac magnetic resonance imaging; RV, right ventricular; RVEF, RV ejection fraction; RVEDV, RV end-diastolic volume; RVESV, RV end-systolic volume.

**Supplemental Figure 1. Example of two-dimensional speckle-tracking echocardiographic analysis to measure RV strain**


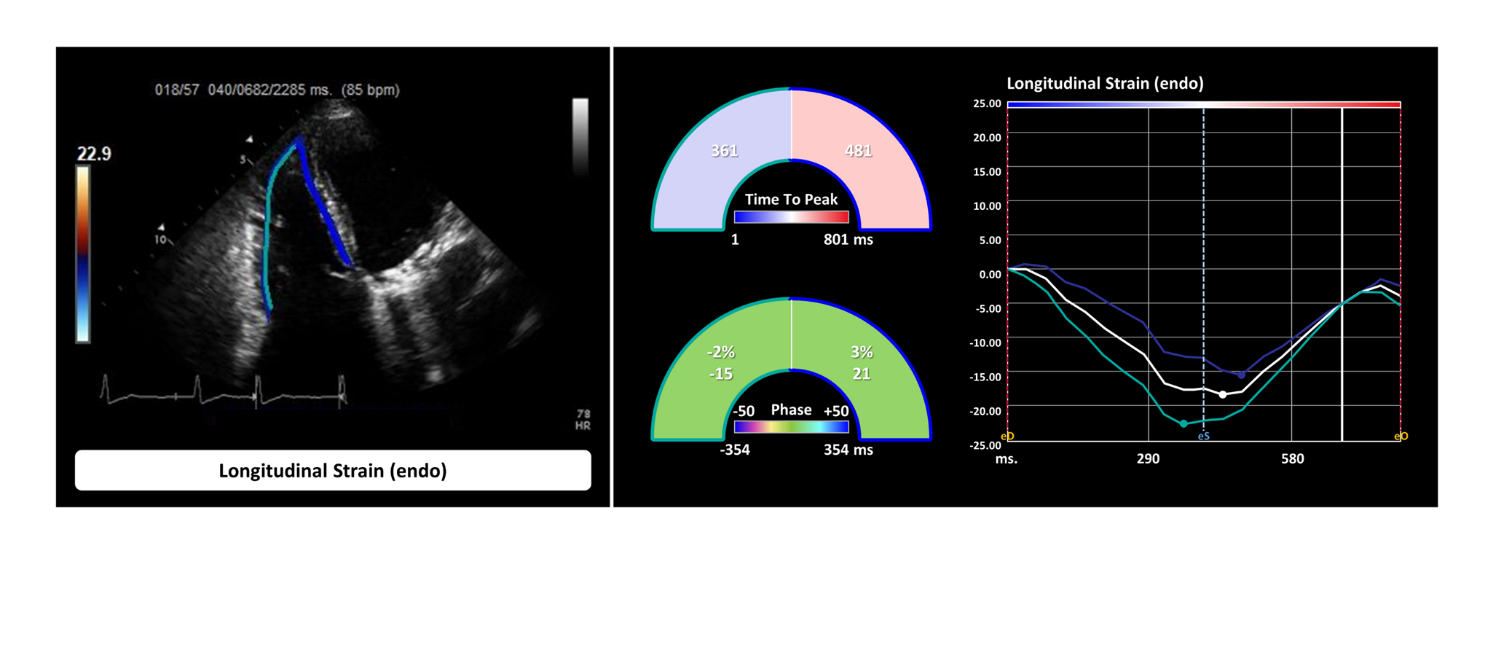


RVFWLS was calculated as the average of the three RV lateral segments (namely basal, mid, and apical), with exclusion of the three septal segments, while RVGLS was calculated as the average of all six segments.

RV, right ventricular; RVFWLS; RV free wall longitudinal strain; RVGLS, RV global longitudinal strain
